# Supplementary material for: Characteristics of Environmental Klebsiella pneumoniae and Klebsiella oxytoca Bacteriophages and Their Therapeutic Applications
Source: Pharmaceutics. 2023 Jan 28;15(2):434. doi: 10.3390/pharmaceutics15020434 (PMC9960720; doi:10.3390/pharmaceutics15020434)
Supplement: Supplementary file 1 [file pharmaceutics-15-00434-s001.zip › Supplementary_Figure_S1.pdf]

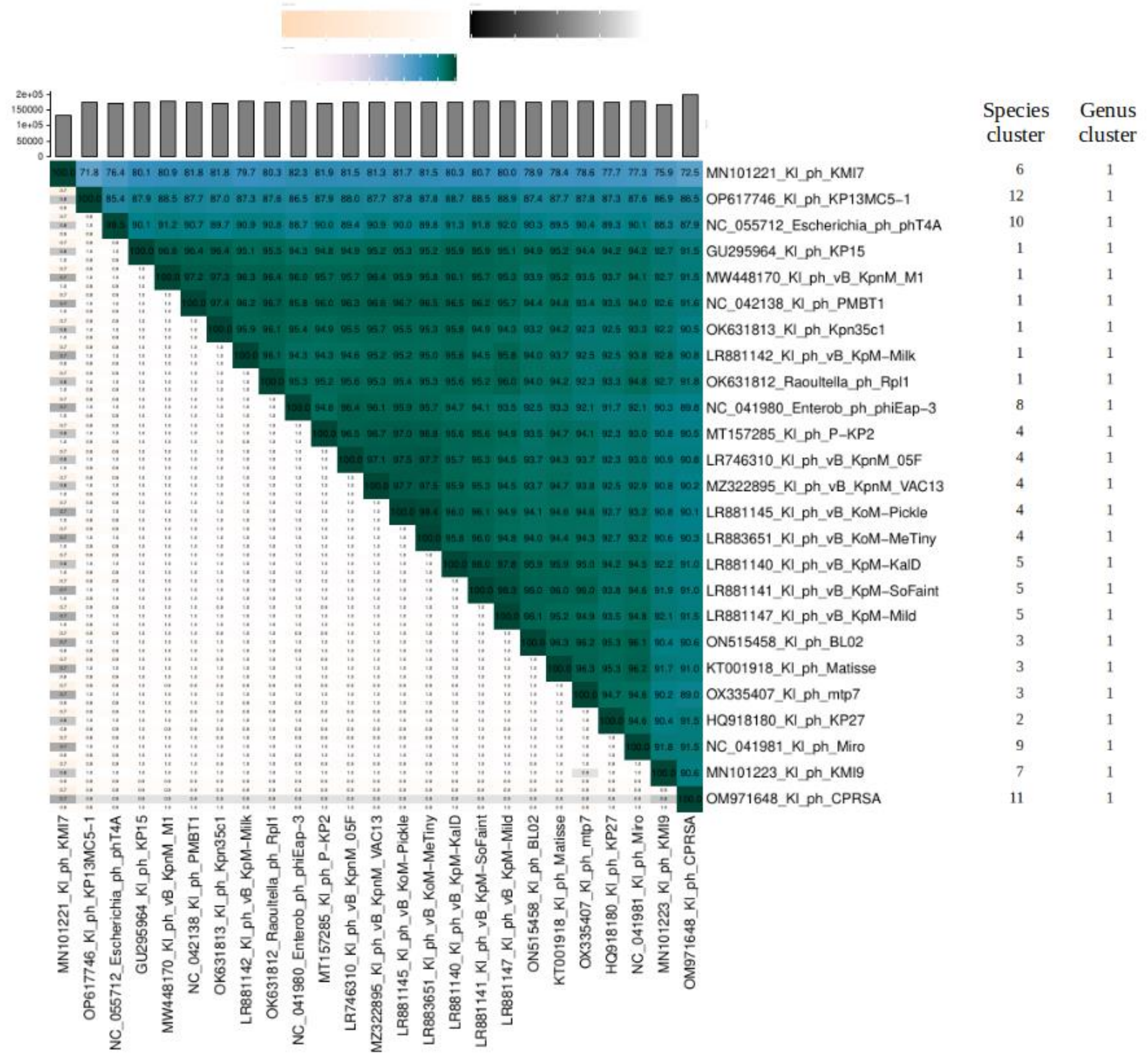

Figure S1. The whole-genome comparison and clustering of phages Rpl1, Kpn35c1, and their closest relatives. The comparison and clustering were performed using Viridic (Virus Intergenomic Distance Calculator; Moraru et al., 2020). Different shades of blue in the right half of the heatmap represent different intergenomic similarities (in %) between the genomes of each pair compared, as indicated above the heatmap and specified by numbers. The left half of the heatmap shows three indicator values for each genome pair: aligned fraction of genome 1 for the genome in this row (top value), genome length ratio for the two genomes in this pair (middle value) and aligned fraction of genome 2 for the genome in this column (bottom value). The darker colors represent lower values as indicated above the heatmap. Phage vB_KoM-Liquor (GenBank acc. no. LR881143) which is 100% identical to vB_KpM-Milk, and CPRSB (OM971649) which is 99.5% identical to phage CPRSA were excluded from the alignment.
