# Supplementary material for: Characteristics of Environmental Klebsiella pneumoniae and Klebsiella oxytoca Bacteriophages and Their Therapeutic Applications
Source: Pharmaceutics. 2023 Jan 28;15(2):434. doi: 10.3390/pharmaceutics15020434 (PMC9960720; doi:10.3390/pharmaceutics15020434)
Supplement: Supplementary file 1 [file pharmaceutics-15-00434-s001.zip › Supplementary_Figure_S2.pdf]

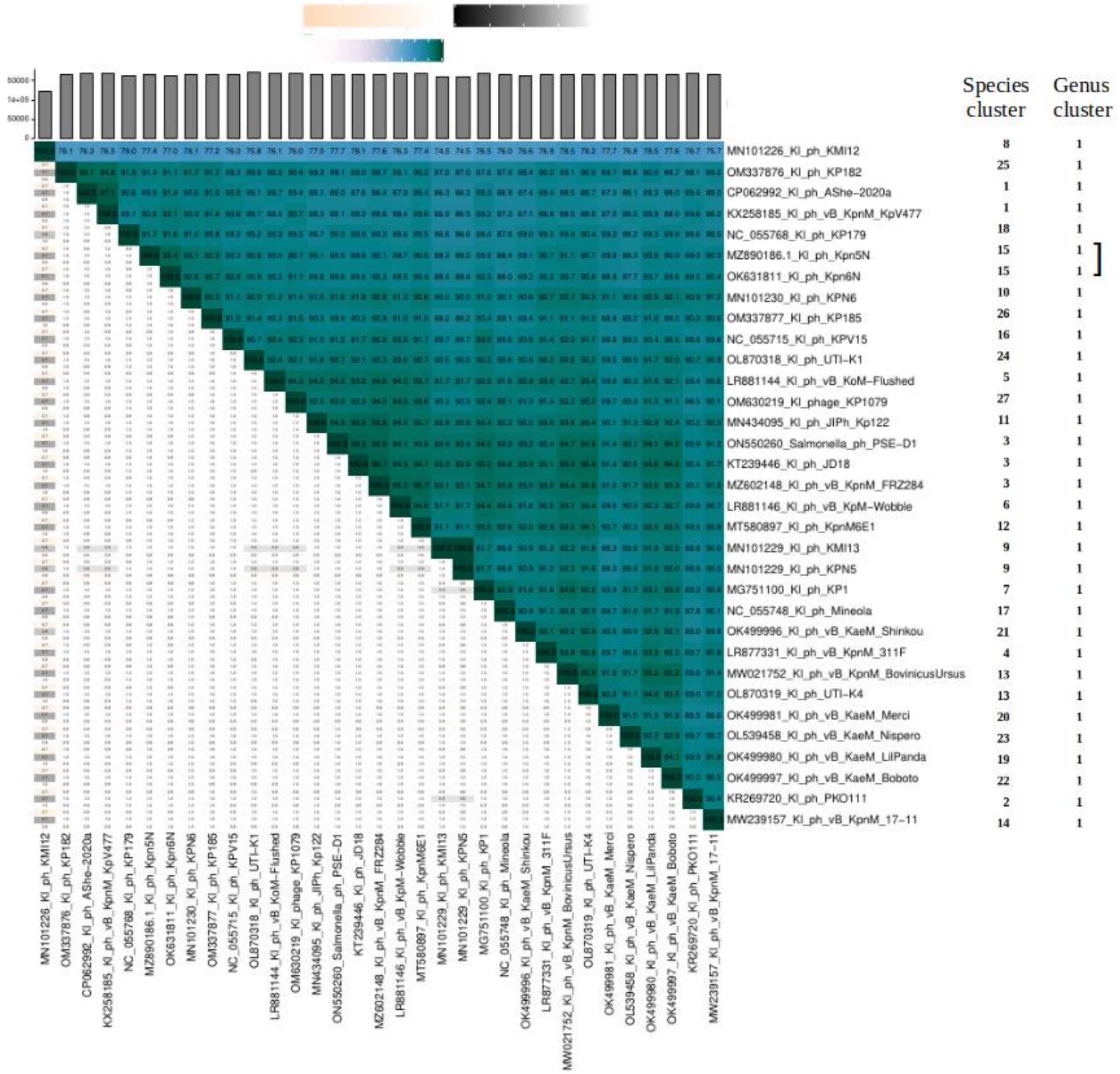

Figure S2. The whole-genome comparison and clustering of phages Kpn5N, Kpn6N, and their closest relatives. The comparison and clustering were performed using Viridic (Virus Intergenomic Distance Calculator; Moraru et al., 2020). Different shades of blue in the right half of the heatmap represent different intergenomic similarities (in %) between the genomes of each pair compared, as indicated above the heatmap and specified by numbers. The left half of the heatmap shows three indicator values for each genome pair: aligned fraction of genome 1 for the genome in this row (top value), genome length ratio for the two genomes in this pair (middle value) and aligned fraction of genome 2 for the genome in this column (bottom value). The darker colors represent lower values as indicated above the heatmap. The proposed new species cluster that includes phages Kpn5N and Kpn6N is indicated by bracket.
