# Supplementary material for: Characteristics of Environmental Klebsiella pneumoniae and Klebsiella oxytoca Bacteriophages and Their Therapeutic Applications
Source: Pharmaceutics. 2023 Jan 28;15(2):434. doi: 10.3390/pharmaceutics15020434 (PMC9960720; doi:10.3390/pharmaceutics15020434)
Supplement: Supplementary file 1 [file pharmaceutics-15-00434-s001.zip › Supplementary_Figure_S4.pdf]

A

```
Kpn5N:   1 MAYTVSIAPLAASAVIGATTNFTATTSGAAAEGTEAFVWTVNGVKQSSVTAAMNYVAAGP  60
           MAY+VSIAPLAASAVIGATTNFTATTSGAAAEGTE FVWTVNGVKQSSVTAAMNYVAAGP
Kpn6N:   1 MAYSVSIAPLAASAVIGATTNFTATTSGAAAEGTETFVWTVNGVKQSSVTAAMNYVAAGP  60

Kpn5N:  61 AGSKTVKVVATVTPAEGEAETAEAETTLTVQNKTMPAITLTLSPTSVSKEIGQSQVVTAD  120
           AGSKTVKVVATVTPAEGEAETAEAETTLTV+NKTMPAITLTLSPTSVSKEIGQSQVVTAD
Kpn6N:  61 AGSKTVKVVATVTPAEGEAETAEAETTLTVKNKTMPAITLTLSPTSVSKEIGQSQVVTAD  120

Kpn5N: 121 VTGAPSGASIAYVWKRGSSVISGQTGKTITLTEPAEANYTLNCEVTVSAADYNPATATKG  180
           VTGAPSGASIAYVWKRGSSVISGQTGKTITLTE AE +YTLNCEVTVSA DYN  TATKG
Kpn6N: 121 VTGAPSGASIAYVWKRGSSVISGQTGKTITLTESAETSYTLNCEVTVSAPDYNNGTATKG  180

Kpn5N: 181 VEVVFTKKTMSGVSVTLTPESITVEQGSDASFKADVIGAPEGASGTYSWTKDGSPVEGST  240
           + V F KKTMSGVSVTL+P S++ E G      A+V+GAPEGAS  Y W +    +EG T
Kpn6N: 181 IAVAFIKKTMSGVSVTLSPTSVSKEIGQSQVVTANVVGAPEGASIAYVWKRGTVVIEGQT  240

Kpn5N: 241 S-TLVIDTSDIGSQVIGVSVEVSAEDYNSVTVTTTGNVTITKR  282 (of 373)
           + T+ I  S   +  +     VSA DYN VTV+   +VTITK+
Kpn6N: 241 AKTITITESAEANYTLNCEATVSAPDYNPVTVSKGASVTITKK  283 (of 468)
```

B

```
Kpn5N:   2 AYTVSIAPLAASAVIGATTNFTATTSGAAAEGTEAFVWTVNGVKQSSVTAAMNYVAAGPA  61
           A T++++P + S  IG +   TA  +GA +  + A+VW       S  T     +
Kpn6N:  97 AITLTLSPTSVSKEIGQSQVVTADVTGAPSGASIAYVWKRGSSVISGQTGKTITLTESAE  156

Kpn5N:  62 GSKTVKVVATVTPAEGEAETAEAETTLTVQNKTMPAITLTLSPTSVSKEIGQSQVVTADV  121
            S T+    TV+  +    TA     +    KTM  +++TLSPTSVSKEIGQSQVVTA+V
Kpn6N: 157 TSYTLNCEVTVSAPDYNNGTATKGIAVAFIKKTMSGVSVTLSPTSVSKEIGQSQVVTANV  216

Kpn5N: 122 TGAPSGASIAYVWKRGSSVISGQTGKTITLTEPAEANYTLNCEVTVSAADYNPATATKGV  181
            GAP GASIAYVWKRG+ VI GQT KTIT+TE AEANYTLNCE TVSA DYNP T +KG
Kpn6N: 217 VGAPEGASIAYVWKRGTVVIEGQTAKTITITESAEANYTLNCEATVSAPDYNPVTVSKGA  276

Kpn5N: 182 EVVFTKKTMSGVSVTLTPESITVEQGSDASFKADVIGAPEGASGTYSWTKDGSPVEGSTS  241
            V  TKKTMSGVSVTLTPESITVEQGSDASFKADVIGAPEGA+G YSWTKDGSPVEGSTS
Kpn6N: 277 SVTITKKTMSGVSVTLTPESITVEQGSDASFKADVIGAPEGATGVYSWTKDGSPVEGSTS  336

Kpn5N: 242 TLVIDTSDIGSQVIGVSVEVSAEDYNSVTVTTTGNVTITKRVAPAPDGELPYIHPLPFRG  301
           TLVIDTSDIGSQVIGVSVEVSA+DY  VTVT TGNVTITKRVAP PDGELPYIHPLPFRG
Kpn6N: 337 TLVIDTSDIGSQVIGVSVEVSADDYTPVTVTKTGNVTITKRVAPEPDGELPYIHPLPFRG  396

Kpn5N: 302 TAYIWCGWWVMDEIQRMTVEGKDWKLDDPDSDYYLHRYTLAKMLDDYPEVDVQESRNGYI  361
           TAYIWCGWWVMDEIQRMTVEGKDWKLDDPDSDYYLHRYTLAKMLDDYPEVDVQESRNGYI
Kpn6N: 397 TAYIWCGWWVMDEIQRMTVEGKDWKLDDPDSDYYLHRYTLAKMLDDYPEVDVQESRNGYI  456

Kpn5N: 362 VHRTALEAGIIY  373 (of 373)
           VHRTALEAGIIY
Kpn6N: 457 VHRTALEAGIIY  468 (of 468)
```

Figure S4: Alignment of amino acid sequences of N-terminal (A) and C-terminal (B) regions of phage Kpn5N and Kpn6N gp17 proteins, conterparts of bacteriophage T4 Hoc.
