# Supplementary material for: Characteristics of Environmental Klebsiella pneumoniae and Klebsiella oxytoca Bacteriophages and Their Therapeutic Applications
Source: Pharmaceutics. 2023 Jan 28;15(2):434. doi: 10.3390/pharmaceutics15020434 (PMC9960720; doi:10.3390/pharmaceutics15020434)
Supplement: Supplementary file 1 [file pharmaceutics-15-00434-s001.zip › Supplementary_Table_S1.pdf]

Table S1. Similarities of phage Kpn35c1 and Kpn6N terminase subunits to terminase subunits of selected model phages

Phage	Terminase subunit	Amino acid sequence motif	E-value	Model of the best matching amino acid sequence	Coverage/ identity (%/%)	Model of similar structure (HHPred)	E-value
Kpn35c1	small	pfam11053 (T4 small terminase sub.)	6.87e-54	T4	90/50	Aeromonas phage 44RR2.8t	9.7e-35
	large	pfam03237 (T4 large terminase sub.)	1.08e-42	T4	97/68	T4	2.9e-58
Kpn6N	small	pfam11053 (T4 small terminase sub.)	1.38e-72	T4	94/81	Aeromonas phage 44RR2.8t	2.7e-34
	large	pfam03237 (T4 large terminase sub.)	1.08e-42	T4	99/83	T4	2.9e-58
