# Supplementary material for: Characteristics of Environmental Klebsiella pneumoniae and Klebsiella oxytoca Bacteriophages and Their Therapeutic Applications
Source: Pharmaceutics. 2023 Jan 28;15(2):434. doi: 10.3390/pharmaceutics15020434 (PMC9960720; doi:10.3390/pharmaceutics15020434)
Supplement: Supplementary file 1 [file pharmaceutics-15-00434-s001.zip › Supplementary_Table_S4.pdf]

**Table S4.** Results of HHpred analysis of selected Rpl1 phage proteins. The best and most relevant hits are shown, including the coordinates of the matching regions, PDB ID and chain identifier, and the HHpred probability (%) in the matched regions.

| Predicted gene product | Protein length (aa) | Matching residues (aa) | HHpred best match | PDB ID | Probability (%) |
|---|---|---|---|---|---|
| **Rpl59_054** | 374 | 22-159 | Protein O-linked-mannose beta-1,2-N-acetylglucosaminyltransferase 1; glycosyltransferase, O-mannosylation, alpha-dystroglycan, TRANSFERASE, SUGAR BINDING PROTEIN; 2.49A {*Homo sapiens*} | 5GGF_C | 99.23 |
| | | 19-177 | Protein O-linked-mannose beta-1,2-N-acetylglucosaminyltransferase 1; glycosyltransferease, O-mannosylation, alpha-dystroglycan, SUGAR BINDING PROTEIN; HET: LEC; 1.211A {*Homo sapiens*} | 5GGN_B | 99.17 |
| | | 224-320 | ENDO-1,4-BETA-XYLANASE Y; CARBOHYDRATE-BINDING MODULE, XYLAN-BINDING, XYLANASE; HET: MSE; 2.1A {CLOSTRIDIUM THERMOCELLUM} SCOP: b.18.1.7 | 1DYO_B | 95.49 |
| | | 224-322 | HYALURONATE LYASE; LYASE, HYALURONAN BINDING CARBOHYDRATE BINDING MODULE, CBM, PL FAMILY 8, PL8, HYL; HET: EDO; 1.2A {STREPTOCOCCUS PNEUMONIAE TIGR4} | 1D0Q_A | 95.12 |
| | | 224-322 | Endo-1,4-beta-xylanase C; Binding Site, Carbohydrates, Enzyme Stability, Substrate Specificity, Endo-1, 4-beta-xylanase, Xylan-binding domain, Thermophilic enzymes, Thermostabilizing Domains, Xylotetraose; HET: XYP; 1.67A {Paenibacillus barcinonensis} | 4XUR_B | 94.02 |
| **Rpl59_056** | 1328 | 1230-1328 | L-SHAPED TAIL FIBER PROTEIN; VIRAL PROTEIN, BACTERIAL VIRUSES, CAUDOVIRALES, SIPHOVIRIDAE, INFECTION; HET: FLC; 2.52A {ENTEROBACTERIA PHAGE T5} | 4UW8_H | 98.80 |
| | | 1231-1286 | Endo-N-acetylneuraminidase; Chaperone, Glycosidase, Hydrolase; HET: TAM, PEG; 2.6A {Enterobacteria phage K1F} | 3GW6_B | 98.46 |
| | | 1231-1386 | Long tail fiber distal subunit; Bacteriophage, Helical sandwich, Tail fiber, polyglycine, VIRAL PROTEIN; HET: MRD, MPD, IMD; 1.70355818561A {Salmonella phage vB_SenMS16} | 6F45_B | 98.53 |
| | | 1233-1321 | Tail spike protein; bacteriophage, tailspike, Acinetobacter baumannii, gp42, VIRAL PROTEIN; HET: MSE; 1.794A {Acinetobacter phage vB_AbaP_AS12} | 6EU4_B | 97.92 |
| | | 1233-1324 | Neck appendage protein; 3-helix bundle, chaperon, CHAPERONE; HET: PEG; 2.2A {Bacillus phage GA-1} | 3GUD_A | 97.80 |
| | | 1185-1328 | Isoform 2 of Myelin regulatory factor; auto-catalytic protease, protein chaperone. trimeric protein, triple coiled-coil, membrane PROTEIN; HET: MSE; 2.4A {Mus musculus} | 7DC3_A | 97.69 |
| **Rpl59_262** | 1032 | 1-1028 | Baseplate wedge protein gp7; T4, baseplate, complex, VIRAL PROTEIN; 3.8A {Enterobacteria phage T4} | 5HX2_A | 100.00 |
| | | 178-448 | Glycosyl hydrolase, BNR repeat; 7-bladed beta-propeller fold, structural genomics, Joint Center for Structural Genomics, JCSG, Protein Structure Initiative, PSI-2, hydrolase; HET: MSE, EDO; 2.2A {Ralstonia eutropha} | 3B7F_A | 70.14 |
| | | 190-417 | Exo-arabinanase; ARABINASE, GLYCOSYL HYDROLASE, HYDROLASE; HET: AHR; 1.04A {Penicillium chrysogenum} | 3A72_A | 67.31 |
| | | 183-417 | oligoxyloglucan reducing-end-specific cellobiohydrolase; BETA-PROPELLER, HYDROLASE; 2.2A {Geotrichum sp. M128} SCOP: b.69.13.1 | 1SQJ_B | 66.14 |
| | | 176-448 | Cellulase; Glycoside Hydrolase Family 74, Xyloglucanase, Xyloglucan, HYDROLASE; HET: XYS, IOD, BGC, EDO; 1.95A {Xanthomonas campestris pv. campestris (strain ATCC 33913 / DSM 3586 / NCPPB 528 / LMG 568 / P 25)} | 7KN8_B | 65.60 |
| **Rpl59_134** | 121 | 5-117 | ENDONUCLEASE II; GIY-YIG, NUCLEASE, HYDROLASE; HET: PEG, MSE; 1.9A {ENTEROBACTERIA PHAGE T4} | 2WSH_A | 99.53 |
