# Supplementary material for: Characteristics of Environmental Klebsiella pneumoniae and Klebsiella oxytoca Bacteriophages and Their Therapeutic Applications
Source: Pharmaceutics. 2023 Jan 28;15(2):434. doi: 10.3390/pharmaceutics15020434 (PMC9960720; doi:10.3390/pharmaceutics15020434)
Supplement: Supplementary file 1 [file pharmaceutics-15-00434-s001.zip › Supplementary_Table_S5.pdf]

Table S5. Results of HHpred analysis of selected Kpn6N phage proteins. The best and most relevant hits are shown, including the coordinates of the matching regions, PDB ID and chain identifier, and the HHpred probability (%) in the matched regions. Results of HHPred analysis of T4 DenA and DenB proteins are shown for comparative purposes.

Predicted gene product	Protein length (aa)	Matching residues (aa)	HHpred best match	PDB ID	Probability (%)
**6N_078**	378	27-162	Protein O-linked-mannose beta-1,2-N-acetylglucosaminyltransferase 1; glycosyltransferase, O-mannosylation, alpha-dystroglycan, TRANSFERASE, SUGAR BINDING PROTEIN; 2.49A {*Homo sapiens*}	5GGF_C	99.07
		27-180	Protein O-linked-mannose beta-1,2-N-acetylglucosaminyltransferase 1; glycosyltransferease, O-mannosylation, alpha-dystroglycan, SUGAR BINDING PROTEIN; HET: LEC; 1.211A {*Homo sapiens*}	5GGN_B	98.97
		227-327	ENDO-1,4-BETA-XYLANASE Y; CARBOHYDRATE-BINDING MODULE, XYLAN-BINDING, XYLANASE; HET: MSE; 2.1A {CLOSTRIDIUM THERMOCELLUM} SCOP: b.18.1.7	1DYO_B	95.63
		227-326	HYALURONATE LYASE; LYASE, HYALURONAN BINDING CARBOHYDRATE BINDING MODULE, CBM, PL FAMILY 8, PL8, HYL; HET: EDO; 1.2A {STREPTOCOCCUS PNEUMONIAE TIGR4}	1D0Q_A	94.87
		227-327	Endo-1,4-beta-xylanase C; Binding Site, Carbohydrates, Enzyme Stability, Substrate Specificity, Endo-1, 4-beta-xylanase, Xylan-binding domain, Thermophilic enzymes, Thermostabilizing Domains, Xylotetraose; HET: XYP; 1.67A {Paenibacillus barcinonensis}	4XUR_B	93.90
**6N_079**	220	28-74	Long-tail fiber proximal subunit; VIRAL PROTEIN, CAUDOVIRALES, MYOVIRIDAE; HET: GOL, PO4, MSE; 1.9A {Enterobacteria phage T4}	5NXF_A	93.90
		30-74	LARGE TAIL FIBER PROTEIN P34; VIRAL PROTEIN, CAUDOVIRALES, MYOVIRIDAE, TRIPLE BETA-HELIX; HET: MSE, GOL; 2.0A {ENTEROBACTERIA PHAGE T4}	4UXE_B	92.61
		104-163	HYALURONIDASE-PHAGE ASSOCIATED; TRIPLE-STRANDED BETA-HELIX, HYALURONAN LYASE, PHAGE TAIL FIBRE, HYDROLASE, GLYCOSIDASE, HYALURONIDASE, SCARLET FEVER; 1.6A {STREPTOCOCCUS PYOGENES}	2WH7_A	69.21
		104-163	HYALURONIDASE-PHAGE ASSOCIATED; LYASE, HYALURONAN LYASE, PHAGE TAIL FIBRE, TRIPLE-STRANDED BETA- HELIX, HYALURONIDASE, SCARLET FEVER; 1.9A {STREPTOCOCCUS PYOGENES}	2WB3_A	44.89
**6N_080**	1535	1431-1530	L-SHAPED TAIL FIBER PROTEIN; VIRAL PROTEIN, BACTERIAL VIRUSES, CAUDOVIRALES, SIPHOVIRIDAE, INFECTION; HET: FLC; 2.52A {ENTEROBACTERIA PHAGE T5}	4UW8_H	98.82
		1431-1535	Endo-N-acetylneuraminidase; Chaperone, Glycosidase, Hydrolase; HET: TAM, PEG; 2.6A {Enterobacteria phage K1F}	3GW6_B	98.77
		1431-1533	Long tail fiber distal subunit; Bacteriophage, Helical sandwich, Tail fiber, polyglycine, VIRAL PROTEIN; HET: MRD, MPD, IMD; 1.70355818561A {Salmonella phage vB_SenMS16}	6F45_B	98.53
		1387-1535	Isoform 2 of Myelin regulatory factor; auto-catalytic protease, protein chaperone. trimeric protein, triple coiled-coil, membrane PROTEIN; HET: MSE; 2.4A {Mus musculus}	7DC3_A	98.05
		1431-1523	Tail spike protein; bacteriophage, tailspike, Acinetobacter baumannii, gp42, VIRAL PROTEIN; HET: MSE; 1.794A {Acinetobacter phage vB_AbaP_AS12}	6EU4_B	97.75
**6N_262**	1032	1-1032	Baseplate wedge protein gp7; T4, baseplate, complex, VIRAL PROTEIN; 3.8A {Enterobacteria phage T4}	5HX2_A	100.00
		179-448	Glycosyl hydrolase, BNR repeat; 7-bladed beta-propeller fold, structural genomics, Joint Center for Structural Genomics, JCSG, Protein Structure Initiative, PSI-2, hydrolase; HET: MSE, EDO; 2.2A {Ralstonia eutropha}	3B7F_A	71.99
		192-417	EXO-1,5-ALPHA-L-ARABINOFURANOBIOSIDASE; HYDROLASE; HET: SO4; 1.6A {GIBBERELLA ZEAE}	2YDT_A	71.83
**6N_057**	138	1-135	ENDONUCLEASE II; GIY-YIG, NUCLEASE, HYDROLASE; HET: PEG, MSE; 1.9A {ENTEROBACTERIA PHAGE T4}	2WSH_A	99.85
**6N_101**	175	36-147	Putative endonuclease; protein-DNA complex, restriction enzyme	2OST_A	95.37

			fold, PD-(D/E)-XK motif, homing endonuclease, group I intron, hydrolase-DNA COMPLEX; 3.1A {Synechocystis sp.}		
		40-91	Holliday junction resolvase Hjc; HYDROLASE; 2.01A {Sulfolobus solfataricus} SCOP: c.52.1.18	4TKD_D	76.4
**T4 DenB***	185	56-160	Putative endonuclease; protein-DNA complex, restriction enzyme fold, PD-(D/E)-XK motif, homing endonuclease, group I intron, hydrolase-DNA COMPLEX; 3.1A {Synechocystis sp.}	2OST_A	95.64
		60-109	Holliday junction resolvase Hjc; HYDROLASE; 2.01A {Sulfolobus solfataricus} SCOP: c.52.1.18	4TKD_D	74.98
**6N_152**	398	1-398	DNA alpha-glucosyltransferase; Transferase; HET: GOL, CME, UDP, EDO; 1.73A {Enterobacteria phage T4}	1XV5_A	100
