# Supplementary figures and images for: Characteristics of Environmental Klebsiella pneumoniae and Klebsiella oxytoca Bacteriophages and Their Therapeutic Applications
Source: Pharmaceutics. 2023 Jan 28;15(2):434. doi: 10.3390/pharmaceutics15020434 (PMC9960720; doi:10.3390/pharmaceutics15020434)

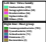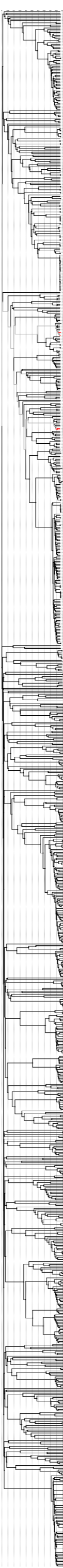

Straboviridae

Terviviridae

Straboviridae

Supplement: Supplementary file 1 [file pharmaceutics-15-00434-s001.zip › Supplementary_Figure_S3.pdf]
